# Subhyaloid hemorrhage as the presenting sign of pregnancy-induced hypertension

**DOI:** 10.4103/0974-620X.57320

**Published:** 2009

**Authors:** Sribhargava Natesh, K Harsha

**Affiliations:** Narayana Nethralaya, Bangalore, India

Dear Sir,

Pregnancy-induced hypertension (PIH) can present with ocular symptoms and signs. Serous retinal detachment, optic neuropathy, retinopathy are some of the known ocular manifestations.[1,2] We report a patient who presented with subhyaloid hemorrhage as the initial manifestation of PIH.

**Figure 1:**
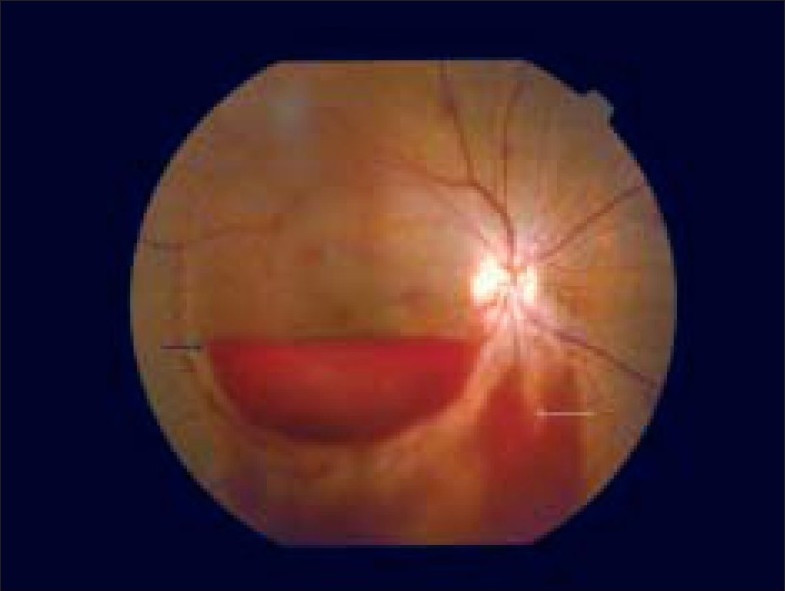
Subhyaloid macular hemorrhage. Note the layered blood (black arrow) and breakthrough bleeding (white arrow)

A 27-year-old primipara presented with sudden and painless decrease in vision in right eye (OD) of 8 days duration. There was no history suggestive of a Valsalva maneuvre. She was not a diabetic or a hypertensive. She was diagnosed as having macular hemorrhage by the primary ophthalmologist and referred to the obstetrician. She also had decreased fetal movements. With a diagnosis of severe PIH, intrauterine growth retardation (IUGR) and abnormal Doppler, emergency cesarean section was done. The baby expired a day after delivery. Patient was seen by us five days after surgery. Best corrected visual acuity was 20/200 OD. Left eye (OS) vision was 20/20. Anterior segment was normal. There was no relative afferent pupillary defect. Fundus OD showed subhyaloid layered, boat-shaped hemorrhage of 10 DD size at the posterior pole [Figure 1] with breakthrough bleeding into vitreous cavity. Peripheral fundus showed multiple, intraretinal flame-shaped hemorrhages. Optic disc and fovea were normal. Fundus OS was normal. Optical Coherence Tomography (OCT) showed a normal contour of the fovea with vitreous detachment at the macula. The hemorrhage was located anterior to the internal limiting membrane and no second membrane was noted superior to the area of layered blood. Electroretinography (ERG) was normal and ruled out a vascular occlusion. Patient did not have features of diabetic, hypertensive or anemic retinopathy. Apart from albuminuria (2+), and raised uric acid level (5.9 mg/dL), rest of the biochemical workup was normal. In four weeks time, she recovered 20/20 vision with resolution of the subhyaloid hemorrhage.

The most common ocular complaint in PIH is blurring of vision, while other relatively less common symptoms are photopsias, scotomas and diplopia. Other ocular manifestations include retinopathy, optic neuropathy, serous detachments and occipital cortical changes, including cortical blindness.[1,2]

Subhyaloid hemorrhage has been described after Valsalva maneuvres, Terson′s syndrome, secondary to arteriosclerosis, hypertension, retinal artery or vein occlusion, diabetic retinopathy, retinal macroaneurysm, chorioretinitis, blood disorders as well as in shaken baby syndrome, age-related macular degeneration and following trauma, chest injury or blunt injury to the eye.[3] Subhyaloid hemorrhage has also been known to occur without any underlying cause. In this patient though fovea was seen hazily through the layered blood, vision was reduced due to the suspended blood.

Subhyaloid haemorrhage treated with Nd:Yag hyaloidotomy has been described in a 22-year-old pregnant woman by Adel and colleagues.[4] Subhyaloid hemorrhage and exophthalmos due to ruptured intraventricular aneurysm in toxemia of pregnancy has been reported by Topilow and Bisland.[5] Although vitreous hemorrhage has been described in HELLP (Hemolytic anemia Elevated Liver enzymes and Low Platelet count) syndrome,[2] this is the first report of subhyaloid hemorrhage as a presenting feature of PIH. Subhyaloid hemorrhage in this case may have been secondary to the acute hypertension.

We conclude that subhyaloid hemorrhage is a rare presenting feature of PIH and ophthalmologists and obstetricians should be aware of the same.
